# Simultaneous Mapping of Multiple Gene Loci with Pooled Segregants

**DOI:** 10.1371/journal.pone.0055133

**Published:** 2013-02-18

**Authors:** Jürgen Claesen, Lieven Clement, Ziv Shkedy, Maria R. Foulquié-Moreno, Tomasz Burzykowski

**Affiliations:** 1 I-BioStat, Hasselt University, Diepenbeek, Belgium; 2 I-BioStat, KULeuven, Leuven, Belgium; 3 Department of Applied Mathematics and Computer Science, Ghent University, Ghent, Belgium; 4 Laboratory of Molecular Cell Biology (Institute of Botany and Microbiology), KU Leuven, Leuven, Belgium; 5 Department of Molecular Microbiology, VIB, Leuven, Belgium; Auburn University, United States of America

## Abstract

The analysis of polygenic, phenotypic characteristics such as quantitative traits or inheritable diseases remains an important challenge. It requires reliable scoring of many genetic markers covering the entire genome. The advent of high-throughput sequencing technologies provides a new way to evaluate large numbers of single nucleotide polymorphisms (SNPs) as genetic markers. Combining the technologies with pooling of segregants, as performed in bulked segregant analysis (BSA), should, in principle, allow the simultaneous mapping of multiple genetic loci present throughout the genome. The gene mapping process, applied here, consists of three steps: First, a controlled crossing of parents with and without a trait. Second, selection based on phenotypic screening of the offspring, followed by the mapping of short offspring sequences against the parental reference. The final step aims at detecting genetic markers such as SNPs, insertions and deletions with next generation sequencing (NGS). Markers in close proximity of genomic loci that are associated to the trait have a higher probability to be inherited together. Hence, these markers are very useful for discovering the loci and the genetic mechanism underlying the characteristic of interest. Within this context, NGS produces binomial counts along the genome, i.e., the number of sequenced reads that matches with the SNP of the parental reference strain, which is a proxy for the number of individuals in the offspring that share the SNP with the parent. Genomic loci associated with the trait can thus be discovered by analyzing trends in the counts along the genome. We exploit the link between smoothing splines and generalized mixed models for estimating the underlying structure present in the SNP scatterplots.

## Introduction

Quantitative traits, such as high ethanol tolerance in *Saccharomyces cerevisiae*, are phenotypic characteristics that vary and are controlled by multiple genetic elements that may contribute differently to the trait. A quantitative trait locus (QTL) is a region in the genome that is linked to the genes that contribute to a quantitative trait. QTL mapping aims at identifying gene loci that determine a specific polygenic trait. This method relies on the extent of co-segregation of genes, which are located in unknown QTLs and that contribute to a quantitative trait, and genetic markers with known chromosomal locations. During a cross between two organisms, the quantitative-trait gene and a genetic marker located on different chromosomes of the same organism will segregate independently and the recombination frequency will be 50%. However, if the marker is located on the same chromosome as the gene, then they could segregate together, depending on the distance between their loci. The closer they are, the lower the recombination frequency, which eventually reaches 0%. This deviation from random segregation, due to the inverse relation between distance and recombination frequency, can be used to identify QTLs.

The advent of new high throughput screening techniques, such as next generation sequencing (NGS), provides a fast way to identify large numbers of single nucleotide polymorphisms (SNPs) on a genome-wide scale. When combined with a pooling of segregants, NGS allows for simultaneous mapping of QTLs throughout the whole genome.

In this paper, we consider analysis of data coming from an experiment, in which the Illumina/Solexa NGS technique [1] was combined with the pooling of segregants. In particular, we apply scatterplot smoothing techniques to identify potential QTLs. We present a semi-parametric approach that uses marker information from a pool of segregants and provides a “smoother based testing procedure” for discovering genomic regions that contain potential gene loci contributing to the phenotypic trait of interest.

## Results

The scatterplot smoothers, Eqs.(1)–(2) and (5), were defined by using a cubic P-spline as basis and a fourth-order difference penalty on the coefficients [2]. They were then fitted to the two pools of segregants. Pool 1 and 2 contained 136 and 31 yeast cells with at least 16% and 17% ethanol tolerance, respectively. For each of these pools three chromosomes have been selected for illustration. The results for the other chromosomes can be found in [3].

### Chromosome XIV

**Figure 1:**
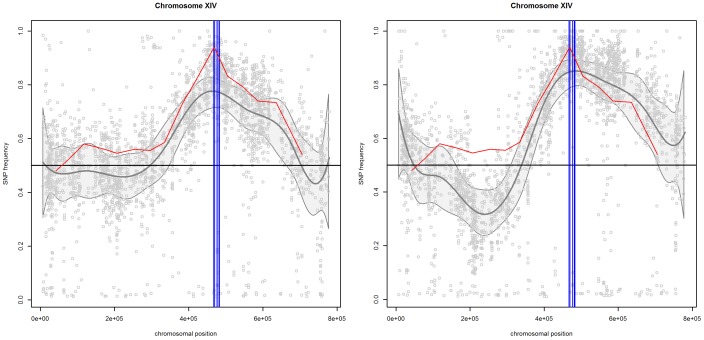
Chromosome XIV. SNP frequencies and smoothed trends for pool 1 (left panel) and pool 2 (right panel). The gray area indicates the 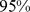 confidence band. The vertical lines indicate the location of the three identified genes, i.e., 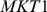, 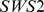 and 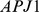. The red line is based on the frequencies of the artificial markers.

Figure 1 presents the scatterplots of mismatch frequencies for the “reliable” SNPs for pool 1 (left panel) and pool 2 (right panel) for chromosome XIV. The plots also include the estimated trends (gray line) with the 95% confidence band (grey area) and the trend of the artificial marker (red line). The two trends follow each other relatively well. The differences observed between both trends is caused by the fact that about 50 individual segregants with artificial markers are used to determine the frequencies, which is different from the BSA process used to generate the SNP frequencies for the ethanol-tolerant segregants.

For both pools, the confidence band indicates a broad region with mismatch frequencies larger than 50%. For pool 2, an additional region with frequencies lower than 50% can be also identified.

The presence of three genes in chromosome XIV, i.e., *MKT1, SWS2* and *APJ1*, has been confirmed by the combination of individual scoring of SNPs with a binomial test and introducing artificial markers at predetermined neutral positions in the genome of the parental strain without high ethanol tolerance [3]. All three genes, located at approximately 470.000 bp, are part of the regions identified by our smoother for both pools.

**Figure 2:**
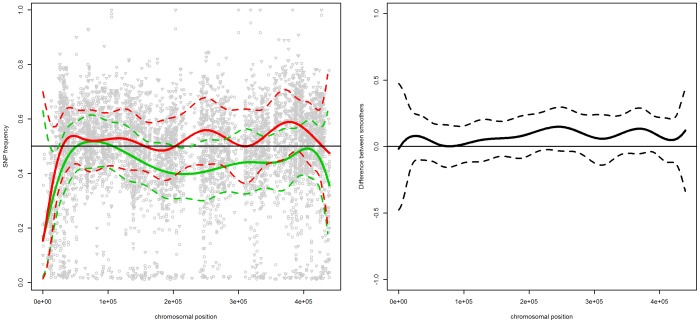
Chromosome XIV. Left panel: SNP frequencies and smoothed trends for pool 1 (circles and green lines) and pool2 (triangles and red lines); right panel: the estimated difference in trends between the two pools. The dashed lines indicate the 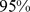 simultaneous confidence band, the vertical lines indicate the location of the three identified genes, i.e., 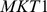, 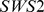 and 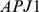.

The left panel of Figure 2 presents the SNP frequencies and smoothed trends for both pools. The right panel presents the smoothed difference between the trends. The difference indicates an enrichment effect in the area around the three QTLs for pool 2. It also suggests an additional effect around 200.000 bp for pool 2 - the SNP frequency drops to approximately 30%. This decrease is also present in pool 1, but it is not as pronounced. This suggests the presence of a minor QTL in the reference strain, which was not present in the strain of the parent with a high ethanol tolerance.

### Chromosome II

**Figure 3:**
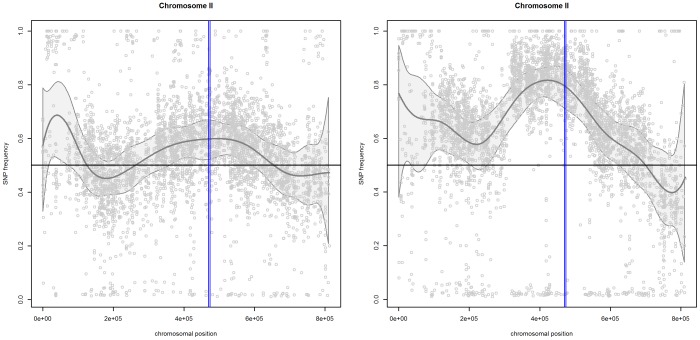
Chromosome II. SNP frequencies and smoothed trends for pool 1 (left panel) and pool 2 (right panel). The gray lines indicate the 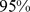 confidence band. The vertical blue line indicates the location of the identified gene, i.e., 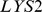.

**Figure 4:**
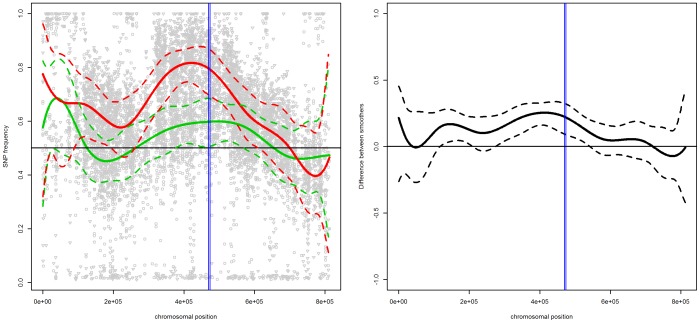
Chromosome II. Left panel: SNP frequencies and smoothed trends for pool 1 (circles and green lines) and pool2 (triangles and red lines); right panel: the estimated difference in trends between the two pools. The dashed lines indicate the 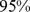 simultaneous confidence band, the vertical blue line indicates the location of the identified gene, i.e., 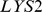.

Figure 3 presents the scatterplots of mismatch frequencies for the “reliable” SNPs for pool 1 (left panel) and pool 2 (right panel) for chromosome II. The plots also include the estimated trends (gray line) with the 95% confidence band (grey area). For both pools, the confidence bands indicate a region with mismatch frequencies larger than 50%. In this region, the presence of one gene around 470.000 bp, i.e., *LYS2*, was confirmed [3]. Note that, for pool 1, the SNP frequencies in the identified region are relatively small, with a maximum around 60%. On the other hand, the frequencies in pool 2 are larger, with the maximum around 80%. This enrichment effect is also identified by the estimated difference between the two pools, shown in the right panel of Figure 4.

### Chromosome IX

**Figure 5:**
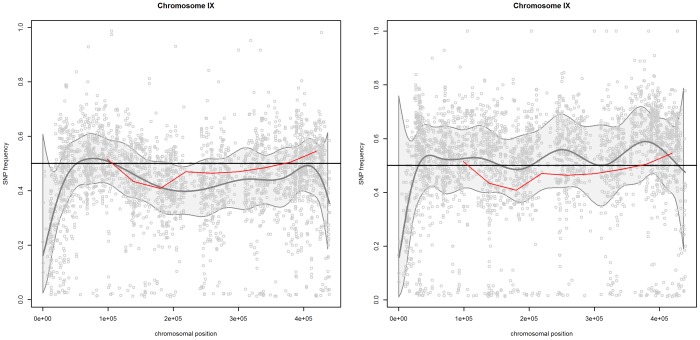
Chromosome IX. SNP frequencies and smoothed trends for pool 1 (left panel) and pool 2 (right panel). The red line is the reference curve based on the frequencies of the artificial markers. The gray lines indicate the 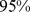 confidence band.

Figure 5 presents the scatterplots of mismatch frequencies for the “reliable” SNPs for pool 1 (left panel) and pool 2 (right panel) for chromosome IX. For both pools, the confidence band does not clearly indicate a region with mismatch frequencies different from 50%. For this chromosome, no QTLs were identified [3].

Note that the reference curve, which reflects the frequencies of the artificial markers, remains below 50% throughout almost the complete chromosome. This anomaly suggests that a SNP frequency of 50% might lead to incorrect results. We propose to conduct a sequencing run on an unselected pool of segregants to estimate the SNP frequency under random segregation. The SNP frequency in the control pool can than be compared with one or more selected segregant pools by using model (5).

**Figure 6:**
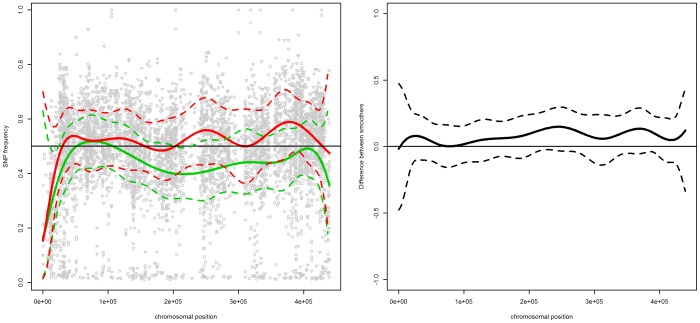
Chromosome IX. Left panel: SNP frequencies and smoothed trends for pool 1 (circles and green lines) and pool2 (triangles and red lines); right panel: the estimated difference in trends between the two pools. The dashed lines indicate the 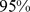 simultaneous confidence band.

The right panel of Figure 6 presents the estimated difference between the two pools. It indicates a significant enrichment effect around 250.000 bp for pool 2.

## Discussion

In our contribution, we showed that scatterplot smoothers are valuable tools for discovering potential QTLs in NGS-BSA experiments. The techniques can be used to model experiments with single as well as multiple segregant pools.

The selected chromosomes, used for validation, illustrate the three possible scenarios one can encounter during genetic mapping, i.e. presence of a major QTL (chromosome XIV), a minor QTL (chromosome II) and the absence of a QTL (chromosome IX). For each of these scenarios, the proposed method performs as expected. Additionally, the possibility to incorporate multiple segregant pools clearly shows to be an important feature. It not only allows investigating enrichment effects, it can also reduce the size of the identified chromosomal regions. Chromosome IX, which does not contain QTLs, also indicates that a control pool is required for estimating the baseline mismatch frequency under random segregation. In the examples, the discovered regions were relatively wide. The low resolution is inherent to the data. For the experiments used here, only the first offspring generation is considered. Hence, the QTLs in the selected segregants are likely to be surrounded by a relative large number of SNP markers. Backcrossing the selected segregants with the parent without the trait will increase the resolution, i.e., the region with flanking SNPs around the QTL will decrease due to the subsequent recombination events. Another possible way to improve the resolution would be to additionally include the information about the first-order derivative of the smoothed trend. The information would allow to identify locations at which the derivative is equal to 0. These would be the locations, at which the estimated SNP mismatch frequency would reach its local maximum or minimum and would thus deviate the most from the random-segregation frequency. This is a topic of further research.

## Materials and Methods

### Data

*Saccharomyces cerevisiae* is the most common yeast strain in industry such as winemaking, baking, and brewing. It is an intensively studied eukaryotic model organisms in molecular and cell biology and has become an important subject for studies in quantitative genetics. The aim of the experiment was to map various QTLs determining high ethanol tolerance in *S. cerevisiae*. For this purpose, a highly ethanol-tolerant yeast strain was crossed with a laboratory strain of a moderate ethanol tolerance. After sporulation, the resulting haploid offspring was screened for high ethanol tolerance, first in a medium with 16% ethanol, and subsequently in a medium with 17% ethanol. The first screening step returned 136 ethanol-tolerant segregants out of a total of 5974 viable haploid yeast cells. The second screening reduced this number further to 31. The difference between 16% and 17% ethanol tolerance is substantial, as 17% ethanol is very close to the maximum ethanol concentration of 18% that yeast cells can tolerate. Most of them will not or only very poorly grow in such a medium. The extent of this difference is also illustrated by the large reduction of viable yeast cells when shifting from a medium with 16% ethanol to the one with 17% ethanol.

After screening, these two pools were subjected to a pooled-segregant genome-wide sequencing analysis by means of high-throughput NGS, as implemented in the Illumina/Solexa NGS technique [1]. The technique measures the fluorescence of PCR-amplified and labeled DNA fragments and translates these intensities into DNA sequences with a length of 40 to 100 basepairs. These millions of overlapping reads are afterwards aligned to a known DNA sequence of the parental laboratory yeast strain (without the trait of interest). Mapping the sequenced reads with SeqMan NGEN 3.0 [4] against the DNA sequence identified many single-nucleotide polymorphisms (SNPs). For each identified SNP, the chromosomal location, the number of sequencing events (reads) for segregant strains, and the number of differences between the segregant strains and the parental strain were retained. The presence of a trait-related gene in the vicinity of the chromosomal location is more likely as the number of deviating SNPs increases.

The number of mapped reads that do not match with the parental reference sequence at the position 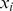 can be interpreted as the number of successes in a group of trials (sequencing events). Hence, we assume that the observed mismatch counts 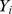 (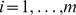) are binomially distributed, i.e.,
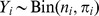
where 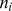 the number of sequencing events and 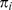 is the probability of the difference between the parental and offspring strain at the chromosomal location 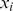.

Note that, Bulk Segregant Analysis [5] was used in this experiment. This method extracts the sequences randomly from a pool of selected segregants instead of applying NGS on each of the selected segregants, individually.

The highly ethanol-tolerant yeast strain was also crossed with 28 partial artificial marked strains [6]. These strains contain several artificial unique sequences of 20 bp with a distance of approximately 20.000 bp between them. After screening the offspring for high ethanol tolerance, the presence of these artificial markers was checked by PCR, for approximately 50 viable segregants in chromosomes VIII to XVI. Moreover, we also had NGS data at our disposal from the parent strain with the trait.

### Filtering

**Figure 7:**
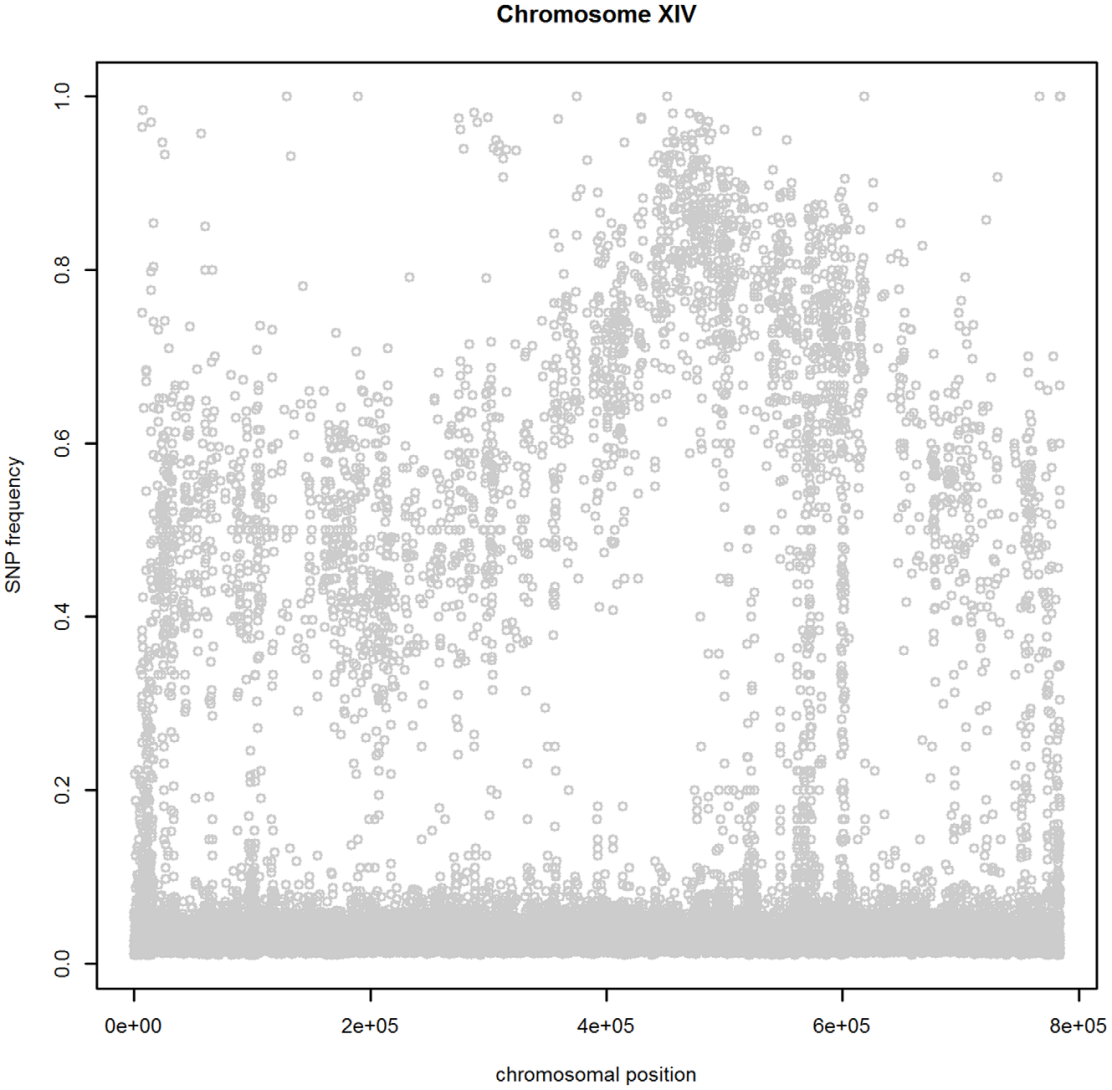
The mismatch frequency for SNPs on chromosome XIV for the segregants with an ethanol tolerance of at least 16%.

There are several issues that have to be taken into account prior to the analysis. The scatterplot of the mismatch SNP frequencies along chromosome XIV, shown in Figure 7, illustrates these issues. First, a large number of SNPs with a mismatch frequency below 10% is observed. The majority of these SNPs probably correspond to sequencing errors. Second, a few high-frequency SNPs are present. These are most likely due to sequencing errors, errors in the reference sequence, or a low number of sequencing events. Third, there is a lot of variability present in the data. This is inherent to BSA-NGS, which relies on the random extraction of the sequences from the pool of selected segregants, i.e. some sequences covering the same SNP location might originate from the same segregant and the number of sequencing events (reads) per SNP location is random.

**Figure 8:**
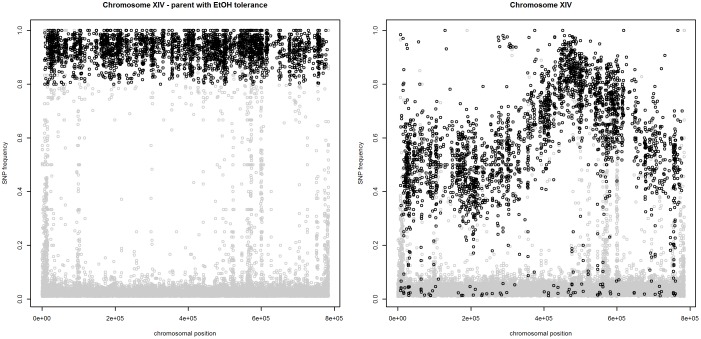
The mismatch frequency of “reliable” (black) and “unreliable” (grey) SNPs on chromosome XIV. Left panel : parental strain with a high ethanol tolerance; right panel: the selected segregants.

To address these issues, one could try to correct the sequencing errors [7], use alternative base-calling procedures [8], [9], or apply filtering. We have chosen for a filtering method, as we did not have access to the raw data necessary for applying the other correction mechanisms. We used the NGS data of the parent with the trait for this purpose and aligned it against the genome of the lab strain without the trait. The differences between both strains were identified and their frequencies plotted against their chromosomal location, as illustrated in Figure 8 (left panel). The plot clearly shows two distinct groups of SNPs with high and low mismatch frequencies. We considered SNPs with a mismatch frequency higher than 80% to be potentially reliable. The second selection criterion was based on the sequencing depth, i.e., the number of reads aligned at a particular location. Dohm *et al.* (2008) [10] showed that a 20-fold sequencing coverage is sufficient to compensate for sequencing errors by correct reads. Hence, we only used the high-frequency SNPs at genomic positions with a coverage of at least 20 reads. In the analysis of the selected pool of segregants, we only consider those SNPs, which we termed “reliable.” The effect of this filtering procedure is displayed in the right panel of Figure 8, and in Table S1.

### Scatterplot Smoother

Trends in the mismatch frequencies for the selected segregants are useful for discovering potential QTLs (see right panel of Figure 8). Non-parametric regression can be used to provide an estimate of the underlying nonlinear relationship between the SNP frequencies and their chromosomal location. The relationship can be expressed as
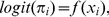
(1)where 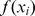 is a smooth function of the chromosomal position. Smoothing splines are commonly used for this purpose. A general spline model of degree 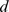 with 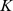 knots can be written as follows:
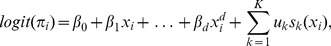
(2)where 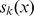 is a set of spline basis functions.

To avoid overfitting, the spline model is typically estimated by considering penalized maximum likelihood estimation, with a penalty term of the form 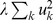. Ruppert *et al.* (2003) [11] showed that the penalized regression problem can be expressed as an equivalent generalized linear mixed-effects model (GLMM):
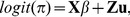
(3)with 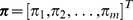, 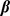 = 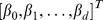, and 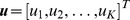. Note that 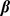 and 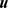 are vectors of the fixed and random effects, respectively, with 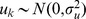 and 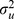 acts as the smoothing parameter. This representation has the advantage that the degree of smoothing can be estimated from the data using standard mixed model software (e.g. Ruppert et al. 2003, chapter 4). The design matrices 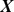 and 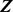 are defined as follows:



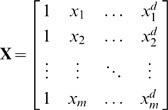
and



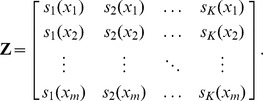



The estimation of the model (3) is performed by means of penalized quasi-likelihood (PQL). Initial estimates for 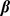 and 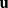 are used to calculate the pseudo-data 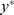:
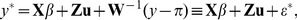
(4)where 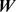 is a diagonal matrix with variances of 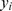 on the diagonal. The pseudo-error 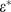 has a variance-covariance matrix 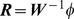, where 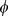 is the dispersion parameter, which is one for the standard binomial model family. Estimating 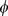, however, allows us to account for overdispersion in the data induced by the large biological variation that typically occurs in BSA-NGS experiments.

Equation (4) resembles a linear mixed-effects model (LMM) formulation for 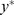. Thus, an LMM is fitted to the pseudo-data, yielding updated estimates of 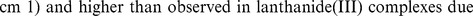, 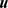, 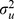, and 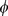. The procedure of calculating pseudo-data and re-fitting the LMM is repeated until convergence.

If multiple segregated pools are available, differences between the observed trends in these pools are useful for identifying potential loci associated with the trait. The scatterplot smoother, proposed in Eqs. (1)-(2), can be extended so as to identify the underlying trends in different pools as well as the pairwise differences between these trends. In particular, we propose
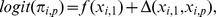
where 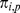 is the probability of the difference between the parental and offspring strain at location 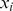 in pool 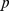, 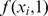 is the scatterplot smoother for pool 1, and 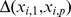 is the difference between pools 1 and 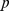 with 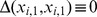 and
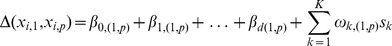
(5)for 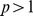. The variance of 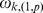 in the GLMM-representation (3) of the model now acts as the smoothing parameter for the difference between the pools.

### Inference

Estimating the underlying trends does not suffice for identifying chromosomal regions that might be linked to the trait. Therefore, we propose a more formal assessment to discover systematic deviations from random segregation for single pools and/or for discovering differences in trends between multiple pools. Our approach is based on confidence intervals or confidence bands for the estimated smoothers.

According to Ruppert *et al.* (2003) [11], an approximate 100(1-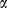)% pointwise confidence band for an estimated penalized spline in the GLMM framework, 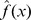, is given by:
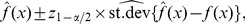
(6)where
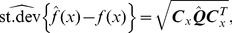
(7)with 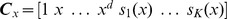 and
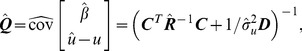
(8)where 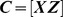 and 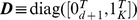


Pointwise confidence bands, however, need to be corrected for multiplicity and ignore serial correlation. Therefore, we propose the use of simultaneous confidence bands, which allow to make joint statements on multiple locations of the fitted curve. A 100(1-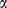)% simultaneous confidence band for 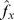 is defined as:
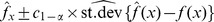
(9)where the critical value, 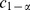, is the (1-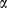) quantile of the random variable



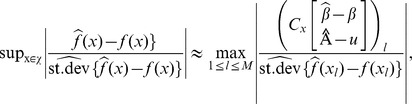
which can be found by simulating from an approximate multivariate normal distribution [11]



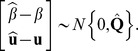



## Supporting Information

Table S1 **Potential and “reliable” SNPs for the three chromosomes in every pool.**
(PDF)
