# Supplementary material for: Simultaneous Mapping of Multiple Gene Loci with Pooled Segregants
Source: PLoS One. 2013 Feb 18;8(2):e55133. doi: 10.1371/journal.pone.0055133 (PMC3575411; doi:10.1371/journal.pone.0055133)
Supplement: Table S1 — Potential and “reliable” SNPs for the three chromosomes in every pool. (PDF) [file pone.0055133.s001.pdf]

Supplementary Material - Table S1

Table S1. Potential and “reliable” SNPs for the three chromosomes in every pool.

	Pool 1 (16% ethanol tolerance)		Pool 2 (17% ethanol tolerance)	
	Potential SNPs	“Reliable” SNPs	Potential SNPs	“Reliable” SNPs
Chromosome II	76639	3995	116722	4151
Chromosome IX	43380	2703	62966	2793
Chromosome XIV	72950	2581	110978	2619
